# Influence of Surface Micro-Patterning and Hydrogel Coating on Colloidal Silica Fouling of Polyamide Thin-Film Composite Membranes

**DOI:** 10.3390/membranes9060067

**Published:** 2019-06-04

**Authors:** Ibrahim M.A. ElSherbiny, Ahmed S.G. Khalil, Mathias Ulbricht

**Affiliations:** 1 Lehrstuhl für Technische Chemie II, and Center for Water and Environmental Research (ZWU), University of Duisburg-Essen, 45141 Essen, Germany; ibrahim.elsherbiny@uni-due.de; 2 Physics Department, and Center for Environmental and Smart Technology, Faculty of Science, Fayoum University, 63514 Fayoum, Egypt; asg05@fayoum.edu.eg

**Keywords:** surface micro-patterning, polyamide thin-film composite membrane, colloidal fouling, silica fouling, stimuli-responsive polymer, water desalination, antifouling propensity, fouling resistant membranes

## Abstract

In this work, colloidal fouling by silica particles of different sizes on micro-patterned pristine and poly-(*N*-isopropylacylamide)-coated polyamide (PA) thin-film composite (TFC) membranes was studied. The competing impacts of surface micro-patterning vs. surface chemical modification on enhancing antifouling propensity in unstirred dead-end filtration conditions were systematically explored. Spatially selective deposition of silica microparticles (500 nm), driven by unequal flow distribution, was observed on micro-patterned membranes such that silica particles accumulated preferentially within the surface pattern’s valleys, while keeping apexes regions not fouled. This interesting phenomenon may explain the substantially enhanced antifouling propensity of micro-patterned PA TFC membranes. A detailed mechanism for spatially selective deposition of silica microparticles is proposed. Furthermore, micro-imprinted surface patterns were revealed to influence deposition behavior/packing of silica nanoparticles (50 nm) resulting in very limited flux decline that was, almost, recovered under influence of triggering stirring stimulus during a continued silica filtration experiment. The current findings provide more insights into the potency of surface micro-patterning consolidated with hydrogel coating toward new fouling-resistant PA TFC membranes.

## 1. Introduction

Membrane fouling, energy demand, and high operating costs are among the main challenges currently faced by water desalination processes using polyamide (PA) thin-film composite (TFC) reverse osmosis (RO) membranes [1]. Fouling is a phenomenon of deposition and/or attachment of retained species on a membrane surface that can deteriorate its performance [2]. In principle, two main fouling phenomena are observed: external fouling (on outer surface) and internal fouling (in membrane pores) [3]. Colloidal fouling, i.e., deposition of retained colloidal and particulate matter, occurs via surface fouling since PA barrier layer is considered to be non-porous [4,5]. The deposited colloidal particles form a cake layer atop membrane surface and, consequently, increase the hydraulic resistance, as well as influence the recovery rate. In certain circumstances, such a cake layer may exacerbate the concentration polarization because of hindering back diffusion of retained solutes, resulting in a significant flux decline [6,7].

Generally speaking, colloidal silica fouling of RO membranes is more severe at high permeate flux values, due to the increased convective particle mass transfer and/or comparatively lower cross-flow velocity [5,8]. Moreover, colloidal fouling rate on flat RO membranes was observed to be not well related to Reynolds numbers (Re) in laminar flow conditions [9]. Only at turbulent flow did it show a dependence on Re because of the improved back transport rate, in particular via shear-induced diffusivity [9]. Therefore, shear rate over the membrane surface may contribute to membrane fouling [6,7,8,10]. Other parameters, e.g., feed concentration and ionic strength, were also investigated [5].

In literature, colloidal silica fouling of many commercial PA TFC RO membranes was extensively examined to find out the impacts of PA characteristic morphology and surface properties, usually employing cross-flow filtration. Generally, the rate of colloidal silica deposition on reverse osmosis membranes is determined by the interplay between double-layer repulsion, and the oppositely acting hydrodynamic force, generated by the convective flow toward the membrane surface (also called permeation drag) [5,6,9]. In case of PA TFC membranes, colloidal silica fouling was found to be dominated by permeation drag because of higher permeate fluxes than in the case of cellulose acetate membranes, where double-layer repulsion plays a significant role [5,11,12]. Higher permeation rates through PA TFC membranes are commonly attributed to their higher surface roughness. Therefore, membrane surface roughness was assumed to be the most important parameter determining fouling propensity of TFC RO membranes [5,8,11,13]. In contrast, colloidal silica fouling experiments for commercial nanofiltration (NF) membranes in turbulent conditions by Boussu et al. revealed that membrane hydrophobicity was the most effective parameter, while membrane surface roughness had only an influence during filtration of small colloidal particles [14].

The Hermia equation, unifying three well-known pore blocking models and the cake filtration mechanism, is frequently exploited to investigate particle fouling mechanisms at constant transmembrane pressure, in dead-end filtrations and early stages of cross-flow filtrations [15]. Colloidal silica fouling of a TFC brackish water membrane (BW30-65) employing dead-end filtration mode in unstirred conditions using silica nanoparticles (~55 nm) was studied by Wang and Tarabara [16]. The cake filtration mechanism was found to be the dominant fouling mechanism that caused the observed flux decline; however, the first 18% of the flux data (initial filtration stage) showed also good fitting with the intermediate pore blocking mechanism [16,17].

Several attempts were made to mitigate colloidal fouling in RO membrane modules. For instance, feed spacers were introduced to enhance hydrodynamics near the membrane surface [18]. They were designed to create flow instabilities (i.e., turbulences) in a laminar flow regime to promote back diffusion and reduce concentration polarization. Nevertheless, spacers were also found to induce particles/foulants accumulation at certain spots (usually expressed as dead zones), which increases the pressure drop along the feed flow channel [19,20]. Therefore, surface micro- and nanopatterned membranes were recently suggested as promising alternatives to induce surface-mixing effects (with or without feed spacers), to reduce concentration polarization and promote antifouling propensity.

Surface hydrodynamics and particle deposition behavior of model foulants on micro- and nanopatterned ultrafiltration (UF) membranes were simulated at different linear velocities within a laminar flow regime, with flow perpendicular to the surface patterns [21,22,23,24]. Surface patterns were emphasized to substantially influence foulants’ deposition behavior such that most of foulants tend to accumulate in the pattern’s lower regions (valleys), where stagnant flow zones (i.e., low shear stress) were formed at low Re [22,23]. Inversely, local wall shear stress was higher at upper regions (apexes) that prohibited attachment of foulant particles. The pattern dimension was also observed to differently influence foulant deposition behavior depending on Re [24]. In parallel, atomic force microscopy (AFM) force–distance curve measurements, developed by Ducker et al. [25], revealed that the potential energy barrier for silica microparticle deposition on a topographically heterogeneous surfaces was lower in comparison with the smoother corresponding surface [26,27]. The potential energy barrier for the attachment of colloidal rigid sphere particles on patterned surfaces was simulated using Derjaguin–Landau–Verwey–Overbeek (DLVO) interaction energy based mathematical models [28,29].

Colloidal silica fouling experiments for patterned UF flat-sheet and hollow-fiber membranes in both dead-end and cross-flow filtration modes were reported [30,31,32,33,34]. Imprinted surface patterns were emphasized to promote antifouling propensity and enhance the critical flux, which were also observed to be influenced by surface pattern dimension, orientation angle between surface patterns, and feed flow direction, as well as operating parameters [30,31]. Furthermore, morphological characterization of some fouled patterned membranes showed an interesting alignment of deposited silica particles along surface pattern grooves, which reflects that surface patterning may have an influence on the packing of crystallized silica particles, a phenomenon known as “graphoepitaxy” [35]. In parallel, Maruf et al. studied scaling propensity of nanopatterned PA TFC membranes using stirred dead-end experiments [36]. The impact of chemical modification combined with the nanopatterning approach on alginate fouling propensity of semi-aromatic PA NF membranes was also recently investigated [37]. Nevertheless, to the best of our knowledge, colloidal silica fouling on micro-patterned PA TFC membranes remains to be investigated. 

In the current work, the deposition behavior of silica micro- and nanoparticles on pristine and hydrogel-coated micro-patterned PA TFC membranes at the early fouling stage was investigated in unstirred dead-end filtration experiments, and compared with corresponding flat membranes. The preparation and chemical modification of micro-patterned PA TFC membranes were reported elsewhere [38,39,40]. Moreover, the impact of switching on stirring, as an added stimulus, on cake layer resistance in the case of micro-patterned and flat surface modified membranes was investigated. Possible mechanisms for silica microparticle deposition atop micro-patterned TFC membranes (i.e., the interaction between silica particles and patterned membrane surface) were discussed.

## 2. Materials and Methods

### 2.1. Materials

Polyethersulfone (PES; Ultrason E6020P; BASF, Germany) and polyvinylpyrrolidone (PVP; K-30; Sigma-Aldrich, Germany) were dried prior to use, while *N*-methyl-2-pyrrolidone (NMP; 99%; Merck, Germany) and triethylene glycol (TEG; 98%; Arcos, Belgium) were employed as received. For PA synthesis, *m*-phenylenediamine (MPD; ≥ 99%), 1,3,5-benzenetricarboxylic acid chloride (TMC; 98%), D(+)-10-camphorsulfonic acid (CSA; ≥ 98%), and *n*-hexane (95%) from Acros, as well as triethylamine (TEA; for synthesis 99%; Merck, Kenilworth, NJ, USA), were used as received. For synthesis of poly(*N*-isopropylacrylamide) (PNIPAAm), monomers *N*-isopropylacrylamide (NIPAAm; stabilized, 99%; Acros), *N*,*N*’-azobisisobutyronitrile (AIBN; 98%; Sigma-Aldrich), and 1,4-dioxane (99.5%; Acros) were employed. The surface modification step was carried out in phosphate buffer solution (pH 7; Bernd Kraft GmbH, Duisburg, Germany). Sodium chloride (NaCl) was purchased from Fluka. For colloidal fouling experiments, microparticles based on silicon dioxide (SiO_2_; 0.5 μm) and LUDOX® TM-50 colloidal silica (50 wt.% suspension in H_2_O; 50 nm) were purchased from Sigma-Aldrich. Nitrogen and argon gasses were purchased from Messer Griesheim GmbH. Ultrapure water, produced by a Milli-Q system (Millipore), was used.

### 2.2. Preparation of Flat and Micro-Patterned PA TFC Membranes

Detailed preparation procedures were established and reported previously [39,40]. Therefore, only a brief description is provided here.

#### 2.2.1. Casting of Flat Isotropic Porous PES Support Membranes

NMP (32 wt.%) and TEG (43.5 wt.%) were mixed thoroughly; then, PVP (12 wt.%) was added to the solution. Afterward, PES (12.5 wt.%) was added, and the obtained dope solution was stirred at room temperature. This dope solution was cast employing a knife with a gap width of 300 µm at a casting speed of 5 mm/s. Thereafter, the polymer film was instantaneously subjected to humid air with a relative humidity of 80% at 22–23 °C for 3 min (exposure time). The obtained turbid film was then precipitated in deionized water and washed.

#### 2.2.2. Preparation of Micro-Patterned Isotropic Porous PES Supports via Micro-Imprinting Lithography

A polydimethylsiloxane (PDMS) mold exhibiting a regular pattern comprising an array of straight parallel grooves, each 30 µm in width and 10 µm in depth at a 12-µm distance between two adjacent grooves, was used for patterning flat PES base membranes employing an adapted custom-made micro-imprinting system [39]. Briefly, the system was assembled and preheated at 60 °C for 40 min. Then, a pressure of 10 bar was introduced using nitrogen gas onto a flat non-porous steel plate placed on the membrane, which on the other side was on top of the micro-patterned PDMS mold. The set-up was kept under such conditions for 35 min.

#### 2.2.3. Preparation of Flat and Micro-Patterned PA TFC Membranes

Flat and micro-patterned PA TFC membranes were prepared employing an interfacial polymerization method [39,40]. The PES support was fixed using specific glass modules [40]. Then, it was immersed in the aqueous monomer solution (2 wt.% MPD, 2 wt.% TEA, and 4 wt.% CSA) for 5 min. The excess reagent was removed by a rubber roll. Thereafter, membranes were immersed in the organic monomer solution (0.1 wt.% TMC in *n*-hexane) for 1 min (reaction time). The obtained composite membranes were thermally cured at 80 °C for 12 min; then, they were finally stored [38].

#### 2.2.4. Surface Modification of Flat and Micro-Patterned PA TFC Membranes

PNIPAAm was synthesized via free-radical polymerization and characterized by attenuated total reflectance (ATR) Fourier-transform infrared (FTIR) spectroscopy (see Section S1, Supplementary Materials). The surface coating procedure of flat and micro-patterned PA TFC membranes using PNIPAAm was established elsewhere [38]; only the well-suited procedure is described here. Briefly, PA TFC membranes were dried overnight. Then, they were fixed using glass modules (see Section 2.2.3). Each dried membrane (19.6 cm^2^) was immersed in 15 mL of PNIPAAm solution (1 mg/ mL) in phosphate buffer at pH 7 and 26 °C for 4 h. The modified membranes were washed and dried [38].

### 2.3. Characterization of Flat and Micro-Patterned Membranes

Membrane surface chemistry and surface charge were examined using Varian Scimitar 1000 FTIR (Varian Inc., Palo Alto, USA) and SurPASS electro-kinetic analyzer (Anton-Paar GmbH, Graz, Austria), respectively; procedures were described elsewhere [38,39]. Membrane surface wettability was measured via the captive bubble method using an optical contact angle goniometer (OCA 15 Plus; Dataphysics GmbH, Filderstadt, Germany). Membrane morphology was scanned using a Quanta 400 FEI SEM (Thermo-Fisher Scientific, Waltham, MA, USA) [38,39]. Membrane surface roughness was quantitatively analyzed by atomic force microscopy (AFM), using a Dimension ICON (Fa. Bruker, USA) [38,39]. Average roughness, *S_a_*, and root-mean-square roughness, *S_q_*, were estimated using NanoScope Analysis 1.5 software.

### 2.4. Measurement of Membrane Separation Performance

Pure water permeability was measured using a 100-mL dead-end stainless-steel cell at ambient temperature and applied pressure of ~14 bar. Separation performance was examined using an NaCl solution of 2000 ppm in water at pH of 6.8, ambient temperature, applied pressure of 14–16 bar, and stirring rate of 700 rpm.

### 2.5. Colloidal Silica Fouling Experiments

Before fouling experiments, pristine and modified membranes were compacted by filtering 1 L of Milli-Q water through each sample (~11.34 cm^2^) employing a cross-flow filtration system at 20 bar [41]. Compacted membranes were stored under water in a lab refrigerator to be later used in the fouling experiments.

Colloidal silica particles with two different sizes (LUDOX^®^ 50 nm, and silica microparticles 500 nm) were utilized for the fouling experiments. Silica feed dispersion (200 mg/L) was prepared by dispersing a predetermined volume of stock silica dispersion in 100 mL of Milli-Q water via vigorous stirring for 15 min and subsequent sonication for 15 min. Subsequently, colloidal silica feed dispersions were characterized with respect to hydrodynamic diameter, d_n_, polydispersity index (PDI), and zeta potential employing a zeta sizer (PSS NICOMP 380 ZLS, Entegris, Billerica, MA, USA), as described in Table 1. The fouling experiments were conducted using a dead-end NF cell equipped with temperature sensor.

#### 2.5.1. Colloidal Fouling Experiments in Unstirred Conditions

Firstly, the dead-end cell was filled with Milli-Q water; then, pure water (i.e., permeate) was collected for 30 min at a constant operating pressure of 16.5–17 bar, and initial water flux was calculated. Afterward, Milli-Q water was replaced by freshly prepared 100 mL of colloidal silica feed dispersion, and the fouling experiment was started at the same pressure and at ambient temperature (23–25 °C) without stirring. The time recording was started at the first permeate drop, and cumulative permeate weight was measured digitally. Colloidal fouling experiments were performed until 96–98 mL of silica dispersion was filtered, if dry fouled membranes were targeted, or until 85–90 mL of silica dispersion was filtered, if wet fouled membranes were needed. Thereafter, fouled membranes were finally stored for further analysis.

During data analysis, several relationships (see Reference [15]) were investigated, including solvent flux decline vs. permeate volume, relative flux decline (Equation (1)) vs. permeate volume, and reverse cumulative flux vs. permeate volume [41]. Furthermore, the applicability of the cake filtration mechanism (Equation (2)) was examined.

(1) $$relative\;flux\;=\frac{flux\;at\;certain\;time\;during\;fouling\;text}{initial\;water\;flux},$$

(2) $$K_{c}V=\frac{2t}{V}-\frac{2}{Q_{0}},$$

where $K_{c}$ is a constant, V is the total permeate volume, t is the filtration time, and $Q_{0}$ is the initial volumetric flow.

#### 2.5.2. Colloidal Fouling Experiments under Influence of Added Stimulus

These experiments were designed to highlight impacts of surface micro-pattering and hydrogel coating on mitigating colloidal fouling of PA TFC membranes via investigating the influence of switching on stirring after a certain time interval of initiating membrane fouling. Colloidal silica nanoparticles, 50 nm, were used as a model foulant. Based on results from previous fouling experiments, the stimulus was triggered when 40 mL of feed dispersion (out of 100 mL) was filtered through the membrane. The experiments were carried out exactly as described in Section 2.5.1; only after 40 mL of permeate was collected, the stirrer was switched on at 700 rpm, before flux recording continued. 

## 3. Results and Discussion

### 3.1. Characterization of Flat and Micro-Patterned Membranes

Surface micro-patterned PA TFC membranes were developed and characterized by our group [39]. Thereafter, adequate surface modification procedures were established [38]. Here, a brief description of the relevant chemical, surface, and morphological characteristics of membranes employed in this study is provided.

ATR-FTIR data for flat and micro-patterned membranes are provided in Section S2 (Supplementary Materials). Several typical IR bands were seen for various functional groups belonging to different membrane layers, i.e., PES, PA, and PNIPAAm. The formation of the hydrogel layer was additionally confirmed using X-ray photoelectron spectroscopy [38]. The results of zeta potential measurements for pristine and surface modified membranes are provided in Table 2. All membranes exhibited an isoelectric point (i.e., pH at which the membrane surface is electrically neutral) in a pH range of 3–4. The zeta potential values at pH 7 for micro-patterned membranes were much less negative than those for flat membranes, i.e., the zeta potential decreased upon surface micro-patterning [39]. Water contact angle values are also presented in Table 2. The micro-patterned membranes showed higher hydrophilicity than the corresponding flat membranes [38,39].

**Figure 1:**
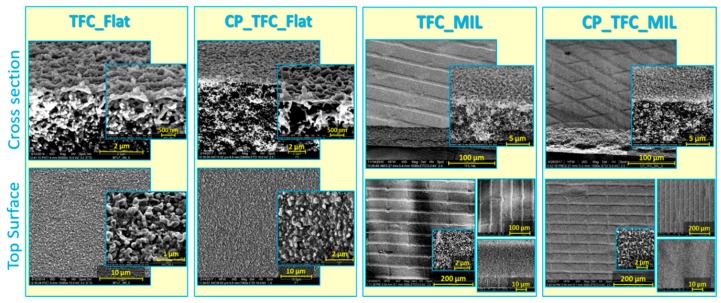
SEM micrographs of top surface and cross-section morphologies at different magnifications for flat vs. micro-patterned surface modified (“CP”) and pristine polyamide (PA) thin-film composite (TFC) membranes.

Surface and cross section morphologies for flat and micro-patterned membranes are introduced in Figure 1. The micro-patterned membranes exhibited a well-defined pattern over the entire membrane surface area even after chemical surface modification. The deviation between the mold geometry and the obtained pattern, i.e., less depth (only 3 µm instead of 10 µm) but slightly wider valleys (15 µm instead of 12 µm), was interpreted by the elastic deformation of the PDMS mold during the micro-imprinting process as previously reported [39]. In addition, surface modified membranes were revealed to exhibit a homogeneous hydrogel coating such that the characteristic “ridge-and-valley” PA morphology was seen to be completely covered. The results of quantitative surface roughness analysis are also presented in Table 2. The average and root-mean-square surface roughness values were significantly higher for micro-patterned membranes than the corresponding flat ones [39]. The hydrogel coating was found to decrease the surface roughness of micro-patterned membranes, which was again interpreted by the covering of the “ridge-and-valley” typical PA morphology [38]. Nevertheless, a very limited increase in surface roughness values was noticed in the case of the flat surface modified membrane (CP_TFC_Flat) compared to the pristine membrane (TFC_Flat) [38]; a similar observation was reported by Yu et al. [42] and Wu et al. [43].

Pure water permeability and separation performance data for flat and micro-patterned membranes are listed in Table 2. It should be noted that a detailed investigation of the merits of micro-patterned PA TFC membranes, over conventional flat ones, with respect to intrinsic separation properties and reduced concentration polarization in dead-end and cross-flow filtration conditions, respectively, was reported elsewhere [39]. The influence of hydrogel coating on pure water permeability and separation performance was also comprehensively discussed before [38]. Briefly, flat and micro-patterned PNIPAAm-modified membranes showed a decrease in both pure water permeability and solution permeability that was attributed to the increase in hydraulic resistance, as a result of coating of an additional layer (i.e., hydrogel) [38]. On the other hand, the coated hydrogel layer, via tight adsorption, was found to increase the membrane selectivity, which was interpreted as a “repairing” mechanism of defects existing in the PA layer [38].

### 3.2. Colloidal Fouling of Flat vs. Micro-Patterned Membranes by Silica Microparticles

The fouling propensity of flat vs. micro-patterned surface modified and pristine membranes by silica microparticles (500 nm) in unstirred conditions was firstly investigated. These particles were chosen as a model foulant due to their relatively big size, which was beneficial for observing direct and clear impacts of surface micro-patterns. Fouling experiments with much smaller silica particles (i.e., 50 nm), comparable to that which frequently exists in natural surface water, were subsequently studied, as outlined in Section 3.3 and Section 3.4.

**Figure 2:**
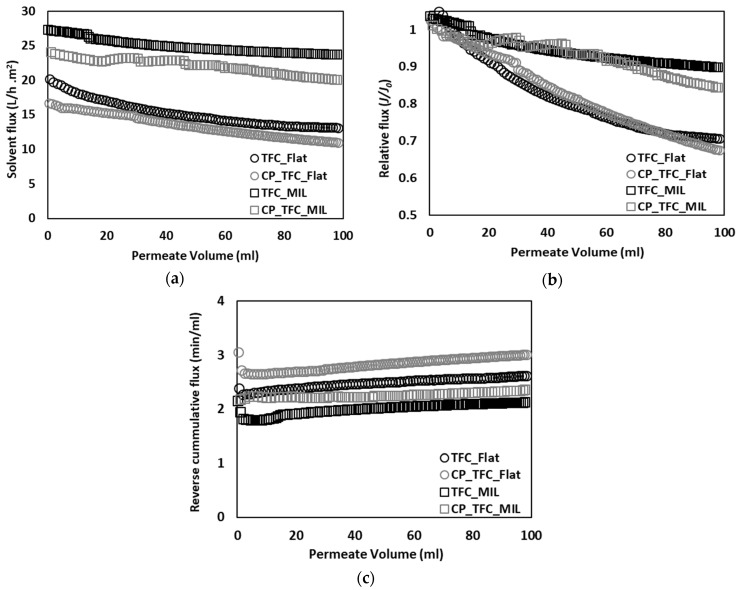
Performance curves for colloidal fouling of flat and micro-patterned membranes by silica microparticles (500 nm) in unstirred dead-end filtration conditions, with constant pressure (~17 bar) and ambient temperature (23–25 °C). The experiments were repeated at least two times, employing a virgin membrane for each; the relative error was <15%. (**a**) Solvent flux decline vs. permeate volume; (**b**) Relative flux decline vs. permeate volume; (**c**) Reverse cumulative flux vs. permeate volume.

Figure 2a presents solvent flux decline plotted vs. permeate volume. The colloidal fouling rate of RO membranes in laminar or stagnant flow conditions was reported to be determined by an interplay between double-layer repulsion and permeation drag [5,11]. Micro-patterned membranes were usually found to enable filtration at higher permeate fluxes than flat membranes by virtue of increased membrane active surface area and enhanced membrane surface roughness [38,39]. As a result, colloidal fouling rate is implied to be dominated by high permeation drag and surface micro-structures [8]. Moreover, TFC_MIL and CP_TFC_MIL (see Table 2) exhibited solvent flux declines of 12.8% and 15.7%, respectively, whereas TFC_Flat and CP_TFC_Flat (see Table 2) showed solvent flux declines of 33.5% and 33.6%, respectively. Consequently, the patterned membranes showed noticeably lower flux reduction compared to their flat counterparts. Nevertheless, surface modified membranes were found to have lower solvent fluxes and slightly higher fouling propensity than pristine membranes.

Relative flux decline was also plotted vs. permeate volume to highlight the difference in the fouling propensity between flat and micro-patterned membranes (see Figure 2b). A substantial improvement in antifouling propensity was observed for the pristine micro-patterned PA TFC membrane (TFC_MIL) in comparison with the flat ones (TFC_Flat), such that a ~50% reduction in the extent of colloidal fouling (estimated by comparing ultimate values of relative flux decline at the end of experiment) was found [41]. This certainly emphasizes the strong impact of surface micro-patterning and subsequent alteration in membrane surface roughness on the colloidal fouling propensity of PA TFC membranes. These findings are consistent with other reported studies [5,8,11,26]. The increase in the fouling resistance of micro-patterned membranes, irrespective of their higher permeability than flat membranes, could be related to irregular flow distribution and modified hydrodynamics atop micro-patterned membrane surfaces (as discussed later). In addition, no significant improvement was observed for surface modified membranes, in comparison with pristine membranes, which reflects the secondary impact of surface modification on the colloidal fouling propensity of PA TFC membranes [8]. Moreover, the applicability of the cake filtration model to the colloidal fouling data was investigated by plotting reverse cumulative flux vs. permeate volume (see Figure 2c). Almost linear relationships were obtained for all fouling systems, indicating that colloidal silica fouling is governed by the cake filtration mechanism [16].

**Figure 3:**
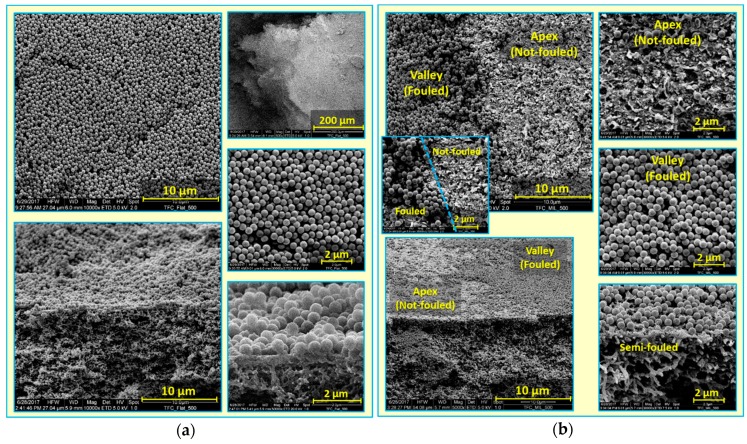
SEM micrographs at different magnifications for colloidal silica fouling of flat (**a**) and micro-patterned (**b**) pristine PA TFC membranes using silica microparticles (500 nm) in unstirred dead-end filtration conditions.

Attempts were made to understand silica deposition behavior, and to explain the substantially enhanced antifouling propensity of micro-patterned PA TFC membranes, despite their higher permeability [41]. Dry, fouled, flat and micro-patterned pristine PA TFC membranes were analyzed using SEM (see Figure 3). A thick, dense and homogeneous silica cake layer covering the entire membrane surface was clearly observed in the case of the fouled TFC_Flat membrane. In contrast, spatially selective deposition of silica microparticles atop the structured membrane surface was found in the case of the fouled TFC_MIL membrane. The majority of silica microparticles were found to deposit in lower regions of surface micro-patterns (valleys) rather than on the upper regions (apexes), where silica microparticles were seen, at higher magnification, to be randomly distributed within the PA ridge-and-valley morphology. Moreover, noteworthy areas of the apex regions were found to be uncovered by silica microparticles (i.e., not fouled during the filtration experiment). Certainly, this interesting phenomenon is limited to the experiment conditions, in particular in terms of the amount of silica microparticles filtered through the membrane. Upon filtering 96% of silica dispersion, about 1.7 mg/cm^2^ (silica amount per membrane sample area) is estimated to be deposited on the membrane surface; at equal flow distribution, this amount corresponds to an average cake layer thickness of 1.1 µm on the TFC_Flat membrane. Considering the pattern dimension of the micro-patterned membrane (TFC_MIL) obtained from SEM (see Figure 1; i.e., apex width of 32 µm, valley width of 15 µm, and valley depth of 3 µm), most silica microparticles seem to be accumulated on about 35% of the membrane sample area (i.e., in valleys) keeping extended areas of the apexes not fouled. These results show explicitly the potential of the imprinted surface micro-patterns on modifying the deposition behavior of foulant particles, and remarkably improving the antifouling propensity, in particular during early stages of filtration. Of course, this behavior would be most beneficial if the particles could be removed from the valleys in a later stage of the filtration (e.g., by enhanced mixing and/or utilizing the properties of the coating; see Section 3.4.)

**Figure 4:**
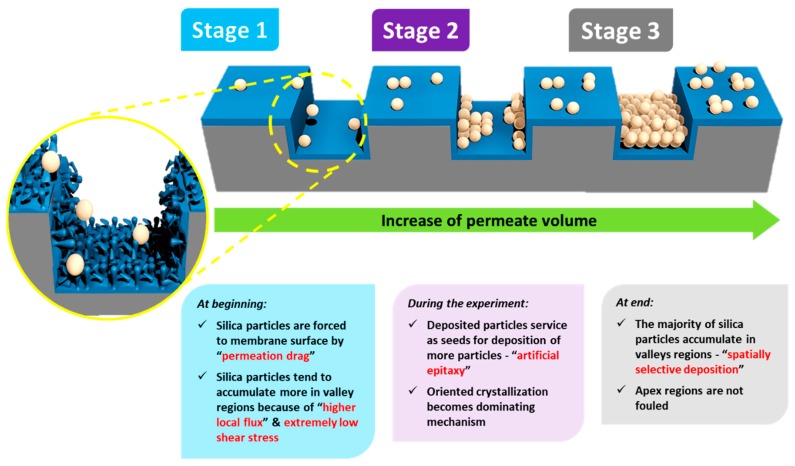
Schematic illustrations for a suggested mechanism interpreting the spatially selective silica microparticle deposition phenomenon on micro-patterned PA TFC membranes in unstirred dead-end filtration conditions.

The influence of surface micro-structures on the particle deposition behavior was simulated and studied experimentally in cross-flow filtration mode, in particular for microfiltration (MF) and UF membranes [22,23,24,32]. Nevertheless, the outputs of these studies, along with the reported work on colloidal silica fouling of flat PA TFC membranes [5,8,11,26], can be specifically consolidated to interpret the significantly enhanced antifouling propensity of micro-patterned PA TFC membranes in unstirred dead-end filtrations. A mechanism is postulated based on filtration results and SEM analysis, with an illustration introduced in Figure 4 [41]. This mechanism comprises three stages.

**Figure 5:**
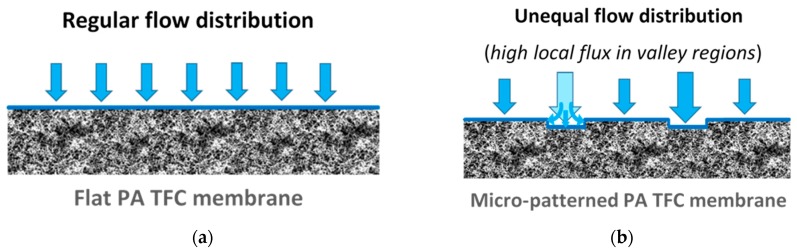
Schematic illustrations for suggested feed flow distribution atop flat PA TFC membrane surface (**a**) vs. micro-patterned PA TFC membrane surface (**b**).

**Stage** **(i)** When the fouling experiment starts, silica microparticles are forced to the membrane surface by permeation drag, caused by high particle concentration in the bulk, as well as high initial flux of micro-patterned PA TFC membranes [8]. Interestingly, more silica particles are directed to the surface pattern’s valleys rather than to apex regions as a result of unequal feed flow distribution near the micro-patterned membrane surface (so-called “local flux”, see Figure 5) and altered surface hydrodynamics, leading to a spatially selective deposition. In general, higher local fluxes are expected in the valleys in comparison with apex regions (Figure 5b). This may be explained by higher specific membrane active surface area, in addition to the higher local permeance because of different PA thicknesses over the surface micro-structures (i.e., much lower PA thickness over valley walls). Furthermore, the surface pattern’s valleys are assumed to act as favorable sites for the deposition of silica microparticles as a result of extremely low shear stress [23]. The valleys in micro-patterned surfaces were simulated in literature to exhibit low DLVO potential energy, called “low-energy pockets” [26,29].
**Stage** **(ii)** Deposited silica microparticles are implied to act as seeds for precipitation of more silica microparticles in a physical process known as “artificial epitaxy”. This scenario, named “orientation by topographic relief”, was previously introduced as a possible mechanism under similar conditions by Givargizov [44,45]. This oriented crystallization of silica microparticles (again favoring spatially selective deposition) is suggested to dominate this stage.
**Stage** **(iii)** At the end, most silica microparticles are deposited within the surface pattern’s valleys, while apex regions are not fouled (at the experiment conditions), which is believed to explain the distinguished antifouling performance of micro-patterned PA TFC membranes.

### 3.3. Colloidal Fouling of Flat vs. Micro-Patterned Membranes by Silica Nanoparticles

**Figure 6:**
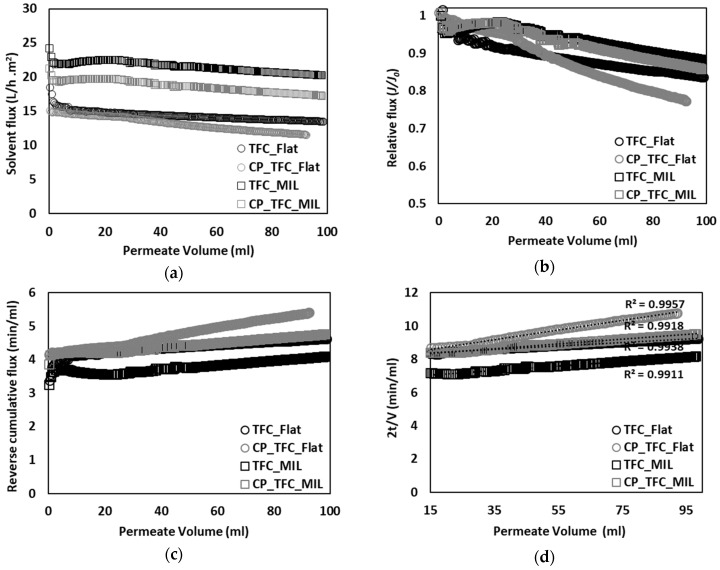
Performance curves for colloidal fouling of flat and micro-patterned membranes by silica nanoparticles (50 nm) in unstirred dead-end filtration conditions, with constant pressure (~17 bar) and ambient temperature (23–25 °C). The experiments were repeated at least two times, employing a virgin membrane for each; the relative error was <15%. (**a**) Solvent flux decline vs. permeate volume; (**b**) Relative flux decline vs. permeate volume; (**c**) Reverse cumulative flux vs. permeate volume; (**d**) Application of cake-layer filtration model to the experimental data.

Colloidal silica fouling (50 nm) of pristine and surface-modified micro-patterned PA TFC membranes was studied in unstirred dead-end filtration conditions and compared with their flat counterparts. Silica nanoparticles, comparable to those often existing in natural surface water and frequently exploited in literature, were employed to investigate the performance of developed micro-patterned PA TFC membranes in conditions closer to real applications. Figure 6a presents the solvent flux decline plotted vs. permeate volume. The differences in initial flux values between Figure 2a and Figure 6a for some membranes exist because of sample-to-sample variations and employing different feed solution. Generally, flat membranes exhibited a typical flux decline as in the case of commercial brackish water membranes [16], whereas very limited solvent flux decline was noticed for micro-patterned membranes. Moreover, the high initial flux of micro-patterned membranes implies that colloidal fouling rate is mainly influenced by permeation drag, as well as morphological membrane surface characteristics, i.e., topography, roughness, and active surface area (as revealed in Section 3.2) [5,8,26].

**Figure 7:**
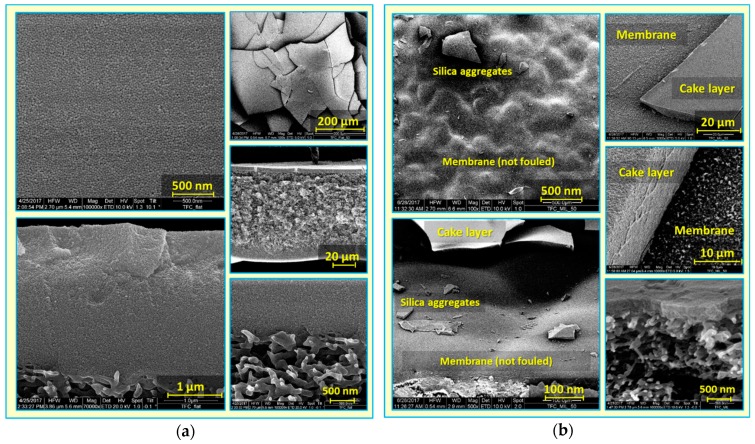
SEM micrographs at different magnifications for colloidal silica fouling of flat (**a**) and micro-patterned (**b**) pristine PA TFC membranes using silica nanoparticles (50 nm) in unstirred dead-end filtration conditions.

Furthermore, relative flux decline was plotted vs. permeate volume (see Figure 6b). Notwithstanding high initial fluxes, a delay in the onset of fouling, accompanied by a lower extent of fouling, was clearly seen for micro-patterned membranes, in comparison with the flat ones. This could be attributed to extended membrane active surface area, as well as enhanced hydrodynamics, as a result of surface micro-patterning [41], which may influence packing of deposited silica nanoparticles [30,35]. Subsequently, dry, fouled, flat and micro-patterned pristine membranes were analyzed using SEM (see Figure 7). SEM micrographs captured at different membrane regions using different magnifications showed very irregular deposition of silica nanoparticles atop micro-patterned membranes, such that silica nanoparticles accumulated or aggregated at certain regions while keeping most of the micro-patterned membrane surface not fouled. In contrast, a homogeneous, crack-free, and thick (~3 µm) silica cake layer was found in the case of the fouled flat membrane. Heterogeneous feed flow distribution (i.e., different local fluxes, see Figure 5) and unequal shear stress in the case of micro-patterned surfaces are believed to influence deposition and subsequent packing of silica nanoparticles. Similar findings were reported for nanopatterned UF membranes during colloidal fouling experiments in cross-flow conditions [30,32]. Moreover, uneven shear stress could also account for the clearly visible cracks in the deposited silica cake layer atop the micro-patterned surfaces. It is worth mentioning that these cracks were also noticed during SEM analysis of some wet fouled membranes (Figure S3, Supplementary Materials), indicating that stress in the silica cake layer should be mainly related to the alteration in silica deposition behavior during the filtration process rather than the procedures for post-filtration analysis [41].

**Figure 8:**
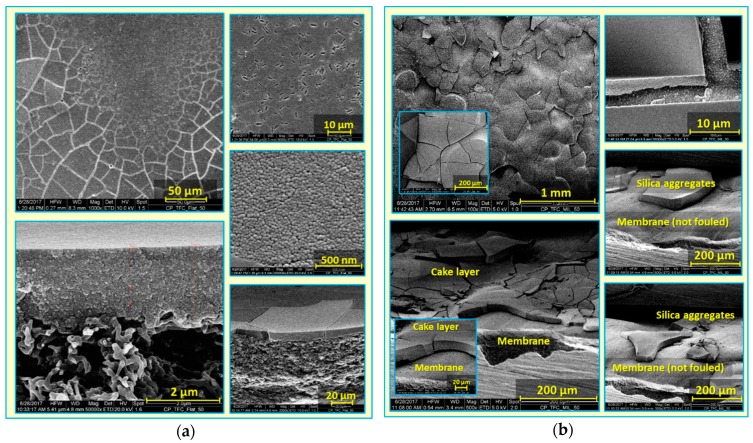
SEM micrographs at different magnifications for colloidal silica fouling of flat (**a**) and micro-patterned (**b**) surface modified membranes using silica nanoparticles (50 nm) in unstirred dead-end filtration conditions.

The impact of hydrogel coating on the colloidal fouling propensity can be deduced from Figure 6b. The extent of silica fouling was higher in the case of surface modified membranes compared to the pristine membranes, despite the decrease in membrane permeability (see Section 3.1). This might be related to the decrease in membrane surface roughness as a result of hydrogel coating. SEM micrographs for dry, fouled, surface modified flat and micro-patterned membranes are shown in Figure 8. Similar silica particle deposition behavior was observed as in the case of pristine PA TFC membranes. Overall, this explicitly emphasizes the consistent potential of the surface micro-patterning approach to mitigate colloidal silica fouling and promote antifouling propensity compared to the surface modification approach.

The applicability of the cake filtration model to silica fouling data for flat and micro-patterned membranes was investigated by plotting reverse cumulative flux vs. permeate volume (see Figure 6c). The fouling data for all PA TFC membranes were found to yield linear relationships; therefore, colloidal silica fouling should be mainly described by the cake filtration mechanism. Subsequently, the fitting range for the cake filtration mechanism was determined by re-plotting the fouling data using the cake filtration equation (see Figure 6d). All colloidal fouling systems were found to exhibit a perfect fitting to the cake filtration mechanism (>99%) beyond a permeate volume of 15 mL (out of 100 mL) [41]. This also implies that, for the PA TFC membranes studied in this work, blocking of the defects in the PA layer does play a role [16].

### 3.4. Colloidal Fouling of Flat vs. Micro-Patterned Membranes by Silica Nanoparticles under Influence of Stirring as an Added Stimulus

**Figure 9:**
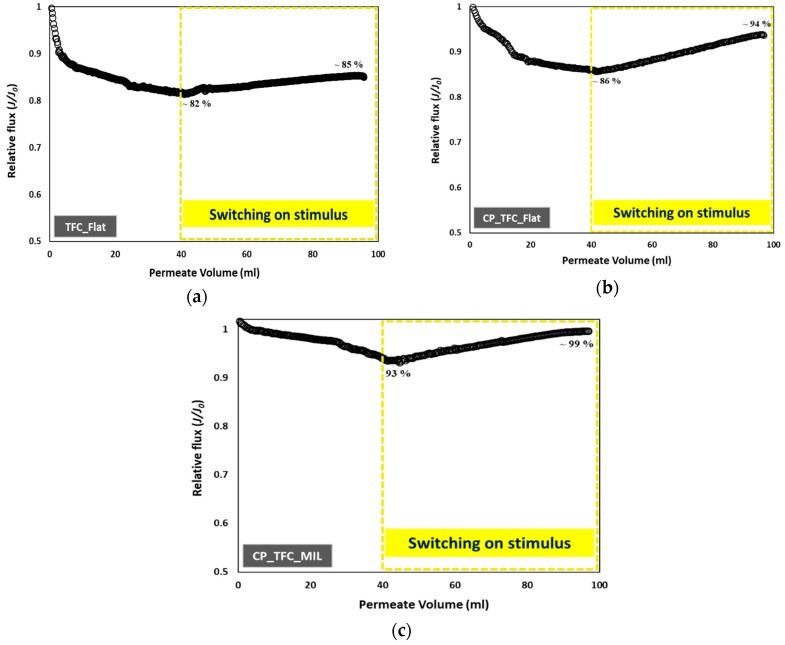
Influence of stirring, as an added stimulus, on colloidal fouling of flat and micro-patterned membranes by silica nanoparticles (50 nm) in unstirred dead-end filtration conditions, with constant pressure (~17 bar) and ambient temperature (23–25 °C). The experiments were repeated at least two times, employing a virgin membrane for each; the relative error was <12%. (**a**) flat pristine TFC membrane (TFC_Flat); (**b**) flat surface modified membrane (CP_TFC_Flat); (**c**) micro-patterned surface modified membrane (CP_TFC_MIL).

The influence of stirring, as an added stimulus, on the silica fouling of three representative membranes was examined. TFC_Flat was selected as the reference sample (i.e., unmodified and not patterned), while CP_TFC_Flat and CP_TFC_MIL were chosen to investigate the impact of hydrogel coating only, as well as synergistic influences of hydrogel coating and surface micro-patterning. The stirring stimulus was described in Section 2.5.2. The filtration period (at which point the stirring stimulus was triggered) was defined based on the examined fitting range of the colloidal fouling data to the cake filtration model (see Section 3.3). As mentioned previously, the cake filtration model was the dominant fouling mechanism in all colloidal fouling systems when a permeate volume of 15 mL was collected. Moreover, in order to ensure formation of the silica cake layer, the stirring stimulus was triggered upon collecting a permeate volume of 40 mL. The results, represented by graphs of relative flux plotted vs. permeate volume, are presented in Figure 9.

Interestingly, triggering the stirring stimulus during the dead-end filtration of silica dispersion was found to restore or recover the solvent flux to significantly different extents, depending on the membrane type. In the case of TFC_Flat, the flux decreased to 82% at a permeate volume of 40 mL due to colloidal silica fouling; then, the flux was partially restored upon triggering the stirring stimulus to reach ~85% at the end of experiment. In the case of CP_TFC_Flat, the flux decreased to ~86% at a permeate volume of 40 mL; thereafter, it was recovered upon stirring to reach ~94% at the end of experiment. On the other side, CP_TFC_MIL showed a very limited flux decline of ~93%, which was completely recovered (~99%) when the stirring stimulus was applied. The main intention of such experiments was to investigate the net improvement in hydrodynamics near the membrane surface when stirring stimulus was combined with surface micro-patterned membranes. This was emphasized for CP_TFC_MIL membranes; this should be related to uneven particle deposition atop this membrane as observed and discussed in Section 3.3. Consolidating the stirring stimulus at a certain point with micro-patterned membrane surfaces is, thus, believed to induce surface mixing effects that increase the shear stress at certain regions of the structured membrane surface and facilitate the detachment of silica nanoparticles [24]. Moreover, irrespective of the secondary impact of surface modification (compared to surface patterning) on colloidal silica fouling, the coated hydrogel layer is also implied to take part in reducing silica cake layer resistance upon triggering the stirring stimulus during continued filtration compared to the pristine membrane.

## 4. Conclusions

The current work supports the earlier findings on the major influence of membrane surface roughness on the colloidal fouling propensity of PA TFC RO membranes. The merits of the surface micro-patterning approach in delaying the onset of colloidal silica fouling and influencing the deposition behavior or packing of silica particles in unstirred dead-end filtration conditions were emphasized. Interestingly, consolidation of micro-patterned membranes and stirring, as an added stimulus, were found to be capable of reversing the flux decline due to colloidal fouling. Although surface chemical modification was found to have a secondary impact on colloidal silica fouling, the coated hydrogel layer was beneficial in suppressing the extent of fouling in the early stage and/or to reduce silica cake layer resistance, when it was combined with surface micro-patterning and a stirring stimulus. Eventually, the outcomes of this study emphasized the potency of surface micro-patterned and optionally hydrogel-coated PA TFC membranes as a competent platform toward sustainable and efficient water purification processes.

## Figures and Tables

**Table 1:** Main characteristics of silica feed dispersions used in colloidal fouling experiments.

Characteristics	Silica Nanoparticles (50 wt.%)	Silica Microparticles (5 wt.%)
Concentration (mg/L)	200	200
pH	7.4	6.7
d_n_ (nm)	45	530
PDI	0.11	0.17
Zeta potential (mV)	−49.2	−50

**Table 2:** Main characteristics and separation performance of flat and micro-patterned membranes employed in this study. IEP—isoelectric point.

Membrane Sample ^1^	Zeta Potential Measurement		Water Contact Angle (°)	Surface Roughness Analysis ^2^		Pure water permeability (L/h∙m^2^∙bar)	Separation Performance	
	IEP	Zeta Potential at pH 7 (mV)		S_a_ (nm)	S_q_ (nm)		Salt (NaCl) Rejection (%)	Solution Permeability (L/h·m^2^·bar)
TFC_Flat	3.8	−80	43.0 ± 5	55	69	1.5 ± 0.1	96.0 ± 0.2	0.90 ± 0.02
CP_TFC_Flat	3.4	−70	58.2 ± 8	58	72	1.1 ± 0.1	97.7 ± 0.2	0.78 ± 0.05
TFC_MIL	3.4	−33	31.0 ± 3	298	366	2.3 ± 0.1	97.0 ± 0.2	1.35 ± 0.10
CP_TFC_MIL	3.8	−26	38.3 ± 6	245	276	2.1 ± 0.1	97.8 ± 0.3	1.26 ± 0.03

^1^ Definition for sample codes: TFC_Flat (flat pristine TFC membrane); CP_TFC_Flat (flat surface modified TFC membrane); TFC_MIL (micro-patterned pristine TFC membrane); CP_TFC_MIL (micro-patterned surface modified TFC membrane).^2^ Surface roughness for patterned membranes estimated using one repeating unit (i.e., apex + valley).

## Supplementary Materials

The following are available online at https://www.mdpi.com/2077-0375/9/6/67/s1: Figure S1: ATR-FTIR spectrum for PNIPAAm homopolymer; Figure S2: (a) ATR-FTIR spectra for flat membranes, (b) ATR-FTIR spectra for micro-patterned membranes; Figure S3: SEM images for wet fouled TFC_MIL membrane by silica nanoparticles (50 nm) in unstirred dead-end filtration experiment.
