# Supplementary material for: Influence of Surface Micro-Patterning and Hydrogel Coating on Colloidal Silica Fouling of Polyamide Thin-Film Composite Membranes
Source: Membranes (Basel). 2019 Jun 4;9(6):67. doi: 10.3390/membranes9060067 (PMC6630248; doi:10.3390/membranes9060067)
Supplement: Supplementary file 1 [file membranes-09-00067-s001.pdf]

membranes

Supplementary Materials: Influence of Surface Micro-Patterning and Hydrogel Coating on Colloidal Silica Fouling of Polyamide Thin-Film Composite Membranes

Ibrahim M.A. ElSherbiny [1], Ahmed S.G. Khalil [2] and Mathias Ulbricht [1,*]

[1] Lehrstuhl für Technische Chemie II, and Center for Water and Environmental Research (ZWU), University of Duisburg-Essen, 45141 Essen, Germany; ibrahim.elsherbiny@uni-due.de
[2] Physics Department, and Center for Environmental and Smart Technology, Faculty of Science, Fayoum University, 63514 Fayoum, Egypt; asg05@fayoum.edu.eg
* Correspondence: mathias.ulbricht@uni-essen.de; Tel.: +49-201-183-3151

Received: 2 May 2019; Accepted: 29 May 2019; Published: date

S1. Synthesis and Characterization of PNIPAAm Homopolymer

Poly-(*N*-isopropylacrylamide) (PNIPAAm) was synthesized by free radical polymerization of NIPAAm monomer in 1,4-dioxane (75.0 wt.%) as solvent using AIBN as initiator (0.3 mol.% relative to monomer). 10.1844 g (9.0×10^{-2} mol) of recrystallized NIPAAm was firstly dissolved in 25 ml of 1,4-dioxane. The reaction solution was degassed and purged with argon gas three times, ~2 min for each time. Thereafter, the reaction flask was preheated up to 60 °C. Then, the initiator solution (0.0445 g, i.e., 2.71×10^{-4} mol, of recrystallized *N,N′*-azobisisobutyronitrile in 4.8 ml of 1,4-dioxane) was quickly added. The reaction was performed at 60 °C under flow of argon gas for 5 h.

Afterward, the solvent was evaporated by rotary evaporator under 105 mbar at 40 °C for about 2 h. The polymer was purified by first dissolving in the lowest possible amount of 3:2 (v/v) acetone/methanol solvent mixture overnight, and then it was recrystallized using diethyl ether as a precipitant by ratio of (1:20) in ice bath. The polymer was dried in a vacuum oven at 40 °C for 3 days and finally stored in lab refrigerator.

The chemical structure of the prepared PNIPAAm homopolymer was characterized using ATR-FTIR spectroscopy (Figure S1). In addition, the number average molecular weight was estimated using gel permeation chromatography (GPC) to be 172.5 kDa at a polydispersity index of 3.26.

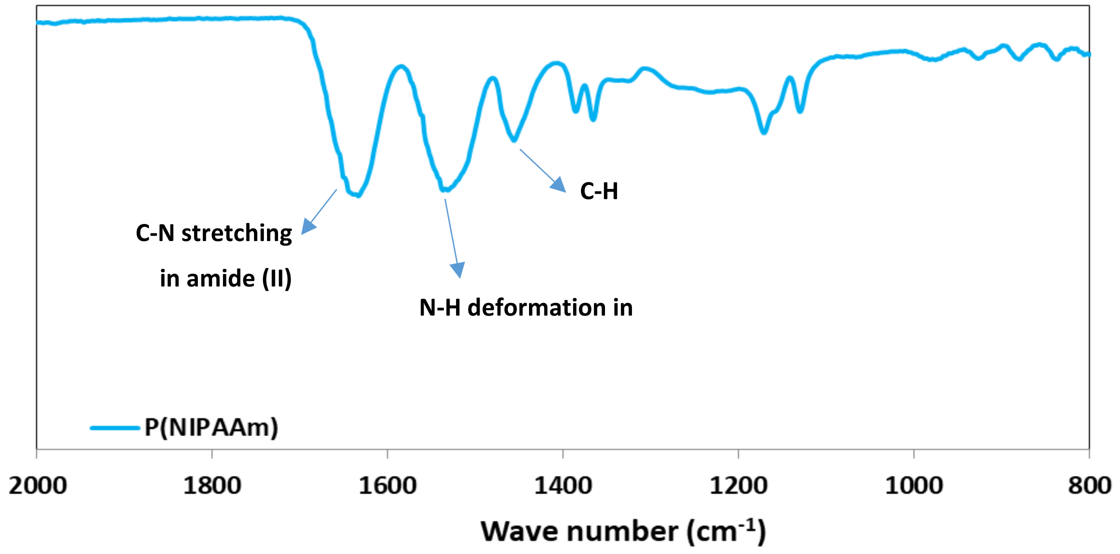

Figure S1. ATR-FTIR spectrum for PNIPAAm homopolymer.

S2. ATR-FTIR Spectra for Flat and Micro-Patterned Membranes

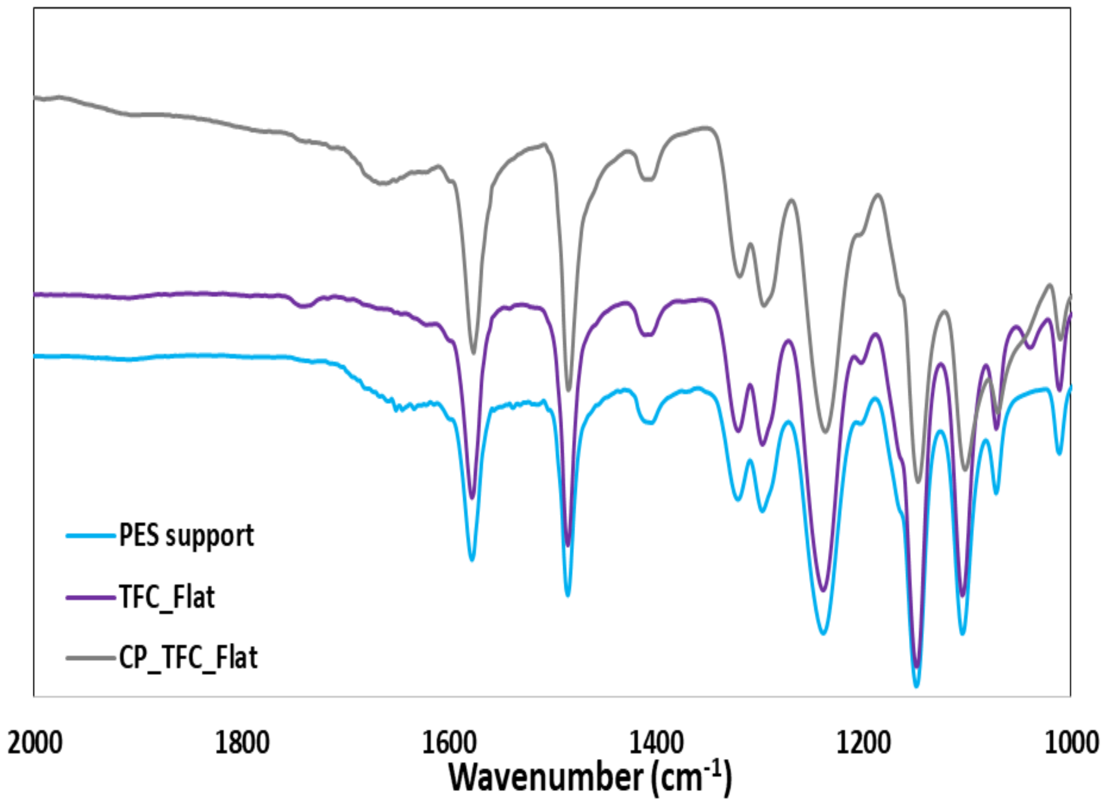

(**a**)

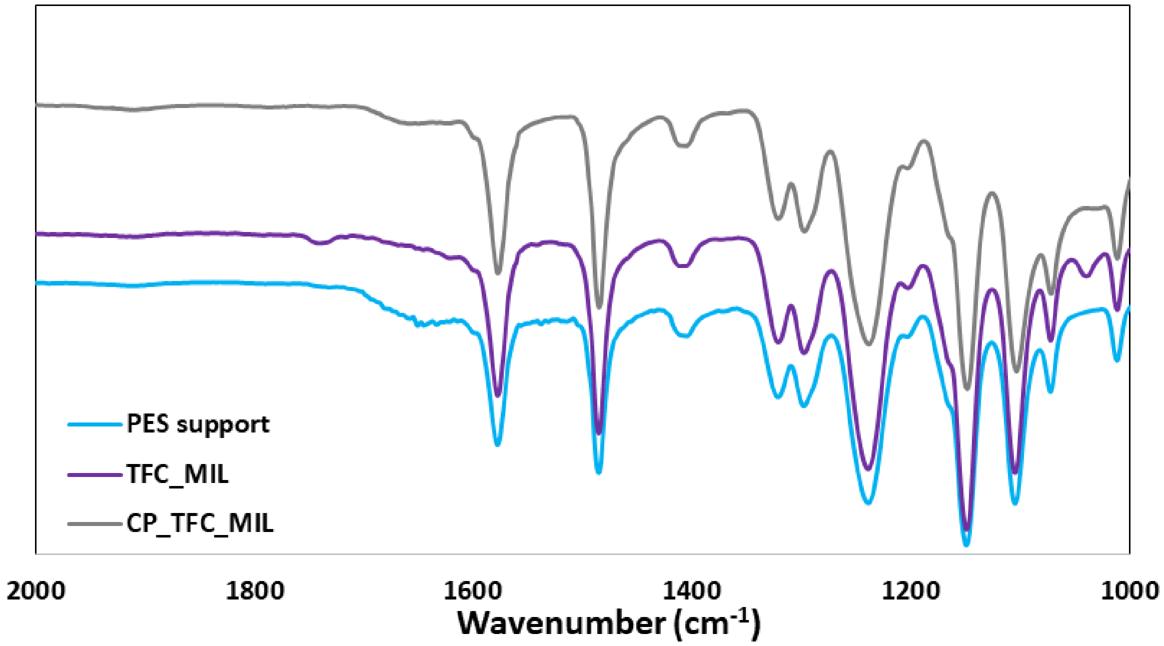

(**b**)

Figure S2. (a). ATR-FTIR spectra for flat membranes; **(b)**. ATR-FTIR spectra for micro-patterned membranes.

S3. SEM for Wet Fouled Pristine Micro-Patterned PA TFC Membrane

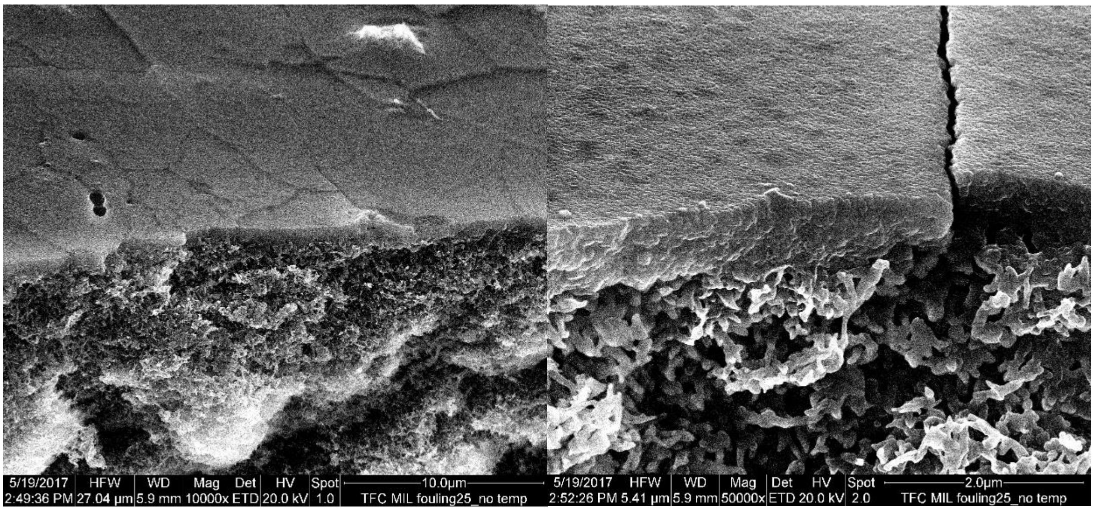

Figure S3. SEM images for wet fouled TFC_MIL membrane by silica nanoparticles (50 nm) at unstirred dead-end filtration experiment.

© 2019 by the authors. Submitted for possible open access publication under the terms and conditions of the Creative Commons Attribution (CC BY) license (http://creativecommons.org/licenses/by/4.0/).
